# An Overview of Bioprocesses Employing Specifically Selected Microbial Catalysts for γ-Aminobutyric Acid Production

**DOI:** 10.3390/microorganisms9122457

**Published:** 2021-11-28

**Authors:** Divakar Dahiya, Jemima V. Manuel, Poonam Singh Nigam

**Affiliations:** 1 School of Human Sciences, London Metropolitan University, Holloway Road, London N7 8DB, UK; ddahiya@hotmail.co.uk; 2 CancerCare Manitoba, 675 McDermot Avenue, Winnipeg, MB R3E 0V9, Canada; jemimavmanuel@gmail.com; 3 Biomedical Sciences Research Institute, Ulster University, BT52 1SA Coleraine, Ireland

**Keywords:** gamma-aminobutyric acid, microorganisms, monosodium-glutamate, biosynthesis, immobilization, genetic-engineering

## Abstract

Gamma-aminobutyric acid (GABA) is an important chemical compound in the human brain. GABA acts as an inhibitory neurotransmitter by inducing hyperpolarization of cellular membranes. Usually, this pharmaceutically important compound is synthesized using a chemical process, but in this short overview we have only analysed microbial processes, which have been studied for the biosynthesis of this commercially important compound. The content of this article includes the following summarised information: the search for biological processes showed a number of lactic acid bacteria and certain species of fungi, which could be effectively used for the production of GABA. Strains found to possess GABA-producing pathways include *Lactobacillus brevis* CRL 1942, *L. plantarum* FNCC 260, *Streptococcus salivarius* subsp. *thermophilus* Y2, *Bifidobacterium* strains, *Monascus spp*., and *Rhizopus spp.* Each of these strains required specific growth conditions. However, several factors were common among these strains, such as the use of two main supplements in their fermentation medium—monosodium glutamate and pyridoxal phosphate—and maintaining an acidic pH. Optimization studies of GABA production were comprised of altering the media constituents, modifying growth conditions, types of cultivation system, and genetic manipulation. Some strains increased the production of GABA under anaerobic conditions. Genetic manipulation focused on silencing some genes or overexpression of *gadB* and *gadC*. The conclusion, based on the review of information available in published research, is that the targeted manipulation of selected microorganisms, as well as the culture conditions for an optimised bioprocess, should be adopted for an increased production of GABA to meet its increasing demand for food and pharmaceutical applications.

## 1. Introduction

Gamma(γ)-aminobutyric acid is considered as a major inhibitory neurotransmitter in the central nervous system (CNS). The action of γ-aminobutyric acid (GABA) is specifically reported in the brain cortex as a neurotransmitter of the CNS. Its key role is reducing neuronal impulsiveness in the nervous system. It was an assumption, mentioned in several reports, that GABA was unable to cross the blood–brain barrier, whereas some studies did show GABA’s ability to cross the barrier. These studies were contradictory, and their methods used in the assessment were not consistent. There has been some evidence in support of GABA food supplements causing a desired calming effect, but again, these were claims made by several consumers online [1]. GABA is an amino acid that is not incorporated in proteins, and therefore, in most cases, it exists as a free amino acid. The natural amino acids are α-amino acids, where the α-carbon, as the central chiral carbon, contains a carboxyl group, a side chain, and an amino group; whereas, GABA is a non-α amino acid due to its structure—the amine group is located away from the carboxylic acid end of the amino acid molecule. Therefore, GABA is not a canonical protein component, as other α-amino acids are. GABA is a γ-amino acid with amine group bonded to the third carbon, mentioned as the γ-position. Studies on this molecule demonstrated its main role to counter the transmission of nervous impulses. Inhibition of neurotransmission is critical in managing conditions of depression, anxiety, and stress.

Besides this, reduced concentration of the neurotransmitter in the brain has been also correlated with Attention Deficit–Hyperactivity Disorder (ADHD) [2] and in neurological irregularities, such as schizophrenia [3]. Other than these, several other pharmaceutical properties of GABA have been reported on non-neuronal peripheral tissues and organs, suitable for its application in cases of diabetes, hypertension, cancer, inflammation, allergy, and in intestinal protection, hepatoprotection and renoprotection. Therefore, GABA has been considered as an alternative potential therapeutic for the prevention and treatment of numerous diseases [4,5,6,7]. The importance of this molecule in the human system has stimulated the need to study its synthesis and its potential sources, which could be used to manage disorders associated with its deficiency, e.g., functional foods, supplemented with GABA. The types of GABA receptors, with their applications in food and pharmaceutical industries, have been described in the following sections. The objective of this review is to summarise the information available on the biosynthesis of this important compound in an economical way, for a commercial scale use in food and pharmaceutical industries.

### 1.1. Types of GABA Receptors

Two types of GABA receptors have been identified and characterized—GABA_A_ and GABA_B_. Each of these receptors has different role in the central nervous system. GABA_A_ is an inotropic receptor, meaning that the binding of GABA alters ion channels, while GABA_B_ is a metabotropic receptor [3]. GABA, as an inhibitory neurotransmitter, causes hyperpolarization of the membrane. Biologically, for a neural transmission to follow, the membrane must be depolarized beyond its threshold. The depolarization stimulates an impulse that is transmitted through the cell. Once it binds to any of the two receptors, chloride ions flow into the cell, a situation that leads to hyperpolarization of the postsynaptic neuron. The described mode of action is applied to manage various mental health problems [1]. GABA(C) receptors (Rs) are also worth consideration as a novel and significant target for the pharmacological treatments of anxiety-related mental health issues, because it does not desensitize, as other receptors GABA(A)Rs, and GABA(C)Rs are desensitized. This is the reported reason that GABA(C)Rs and GABA(A)Rs work differently in the modulation of associative plasticity in LA neurons of rats. GABA(C)Rs have extended the understanding of GABA receptors in fear memory gain and its storage and, therefore, it has been recommended as a possible novel target for the treatment of disorders of anxiety and fear [6].

### 1.2. Properties and Applications of GABA

GABA helps in increasing protein synthesis, increasing the production of growth hormones, and reducing the risk of lungs adenocarcinoma. It is reported to be an effective tumour suppressor for small airway-resulting lung adenocarcinoma [7]; research outcomes have suggested that marker-guided action of GABA, or a GABA(B)R, could be a novel targeted approach for the prevention of pulmonary adenocarcinoma in smokers. GABA has been suggested as an inhibitor in nicotine-imposed stimulatory challenge of non-small cell lung carcinoma in xenograft models [8,9]. GABA is an amino acid that has established its applications in both the food and pharmaceutical industries [10,11]. In addition, GABA has been reported showing antidiabetic, hypotensive, and diuretic effects [1,2,12]. As a neurotransmitter, it helps in improving memory, pain moderation, and regulation of lipids levels in the body. Other researchers have also reported that GABA helps in improving metabolism [12]. Currently, food manufacturers have been adding its small amounts to food preparations, such as shochu, cheese, gammalone, and gabaranone tea [11].

With the increased understanding of the importance of GABA in neuroprotection, asthma, and immunological disorder, the demand for food fortified with this compound has increased and some commercial GABA products (Table 1) are already available in market as supplements.

## 2. Processes of GABA Production

Methods used for GABA production could be chemical or biological processes. The main factor determining a suitable method is the overall economics of the production, which count towards the cost of production and the net output yield that is achieved in selected process. For a commercially viable process, the most economically favourable method of synthesis should be used.

### 2.1. Bioprocesses

In recent years, several reports have been published on the production of medically important GABA using selected microorganisms as effective biocatalysts. Although microorganisms are mostly known for causing diseases and affecting the overall life of human beings, in extensive research, several of them have been identified as non-pathogenic. The GRAS (generally regarded as safe) microorganisms are currently used in the production of various industrially and medically important compounds. The opening of advanced molecular and biotechnology techniques has helped in understanding the growth and metabolism characteristics of selected microorganisms. Besides this, new techniques are currently used to modify the genetic makeup of desired microorganisms to optimize the production of selected secondary metabolites. GABA is one of such metabolites that can be effectively produced by microorganisms [10].

The advantage of using microorganisms in the biosynthesis of an added-value compound is that they could be easily manipulated, and the process could be optimized for the purpose. Therefore, several biological processes employing microorganisms in fermentation systems have been studied for the synthesis of added-value secondary metabolites. A large number of microbial metabolites are utilized in healthcare as antimicrobial, antiparasitic, antitumour, and immunosuppressive agents [13,14]. Few species of microbes are known to produce useful metabolites for the food and fermented beverages industry, these strains could be easily managed diverting them for the biochemistry of GABA synthesis. Due to the deployment of selected microorganisms in the synthesis of this useful compound, there is a need to evaluate their cultivation in fermentation systems and optimize those factors affecting its biosynthesis in culture medium. The aim of such studies has been to establish an optimized cost-effective bioprocess for the production of GABA [10].

The study on the production of microbial metabolites has opened up avenues for the modification of respective microorganisms for optimal production. Therefore, to achieve improved yield of a desired secondary metabolite, growth conditions of microbes may be manipulated to prolong the stationary phase in the culture’s growth cycle. Similar approaches can also be applied for GABA production through microbial fermentation with the support of an inducer monosodium glutamate (MSG) [15]. The selected form of GABA, either natural or synthetic, showed the tendency of receptor binding, and produced effects of inhibition of stress signals and decreases in sleep latency [16]. These outcomes emphasized the need for finding specific microbial strains and studying their capability for biosynthesis of GABA. For this purpose, specific groups of strains were first screened for their ability to produce this molecule, then their physiological requirements were studied for their optimal growth [17]. The characterization of potential microorganisms was followed by the proposition of an optimized biosynthesis process, for cost-effective, commercial-scale production.

### 2.2. Strategies for Biosynthesis

**Figure 1:**
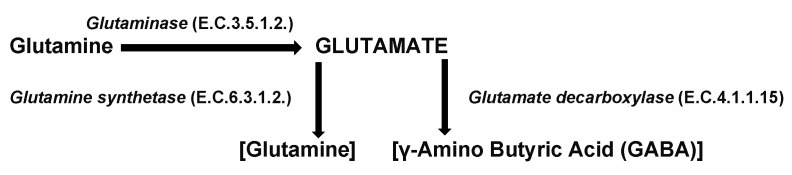
The biosynthesis production process, a non-essential amino acid glutamate is readily decarboxylated to GABA, catalysed by an enzyme glutamate decarboxylase (GAD), EC 4.1.1.15.

When comparing the two methods, microbial production seems to be more promising than chemical synthesis, due to the high biocatalytic efficiency of selected microbial strains. Microbial production of GABA has been studied for optimization of culture conditions needed by specific strains, primarily with relation to growth temperatures, and the time phase of compound appearance in culture medium [17,18,19]. In a biosynthesis production process, a non-essential amino acid glutamate is readily decarboxylated to GABA, catalysed by an enzyme glutamate decarboxylase (GAD), EC 4.1.1.15, as shown below Figure 1:

GAD enzyme is responsible for the decarboxylation of glutamate to GABA and CO_2_, and pyridoxal phosphate (PLP) is used as a cofactor for the activity of GAD. The molecular reaction is summarised as follows:

HOOC-CH_2_-CH_2_-CH(NH_2_)-COOH → HOOC-CH_2_-CH_2_-CH_2_NH_2_ + CO_2_ (1)

GAD in mammals exists in two isoforms with molecular weights of 67 and 65 kDa, reported as GAD67 and GAD65, which are encoded by two different genes on different chromosomes GAD1 and GAD2 genes.

Few fermentation methods for the bioproduction have been tested and proposed [20,21,22,23,24,25]. Some of these have reported the use of immobilized microbial cells, batch fermentation, and sourdough fermentation. The immobilized cell method is a technique mostly applied in situations where excessive free cell mass can have an adverse effect on the production of the required metabolite. Therefore, the microbial cells are immobilized to prevent surplus cell division, channelizing the energy from carbon source for the production of metabolite [18,19,20].

Besides the changes in microbial-cultivation methods, the modifications in the energy sources used for the growth of microbes in fermentation medium were also studied. The productivity of a metabolite could be altered, when wheat flour, a carbon source, was replaced by amaranth and quinoa flours [26]. Varying carbon sources is an effective technique to maximize the yield of a metabolite in the production line. The strains could be acclimatised to metabolize readily available carbon sources as low-cost raw materials for economical production [17,18].

The changes noted in the production of GABA, by reducing the cost of process and increasing the yield, are encouraged by the increasing demand for this compound in food and pharmaceutical industries.

## 3. Selected Microbial Strains

Manufacturers seek to identify the best methods to improve production to maximize their profits. Therefore, some strains of microorganisms used in the food industry have also been studied for their effectiveness in GABA production. The modification in microorganisms’ metabolic pathways directs the energy towards the production of the target compound. The stationary phase in the microbial fermentation process is characterized by the accumulation of secondary metabolite, which could be toxic to microorganism. The toxicity stimulates the onset of death phase, where some modifications in culture parameters, such as pH and temperature, can help the microorganism build tolerance for metabolites produced in fermentation. Modified growth environments of incubation under anaerobic condition have been successful in producing a higher yield of GABA in fermented products by fungal cultures [27]. A new source of GAD to produce high yields of GABA has been studied in engineered microorganisms, like *Escherichia coli* [28].

Concise information on specifically selected GABA-producing strains is discussed in following sections.

### 3.1. Lactic Acid Bacteria

#### 3.1.1. Production and Factors

Studies have been performed to test the ability of specific strains of lactic acid bacteria (LAB) to produce GABA. Two LABs, selected for their capability to produce a significant amount of metabolite, were *Lactobacillus* *brevis* CRL 1942 [18] and *L. brevis* GABA *057* [20]. Among these, *L. brevis* CRL 1942 was reported to be more efficient in production. During the fermentation process, LAB strains were provided with suitable carbon sources in medium and supplemented with MSG, a commercially available product, mostly used in the food industry. Results indicated a significant concentration of GABA produced in *L. brevis* CRL 1942 within a short period of fermentation (50 mM in 96 h). The process parameters optimized for this LAB included MRS broth, quinoa, and MSG, and the incubation temperature of fermentation was set at 30 °C [18]. This study demonstrated that the microbial synthesis could be more cost effective after the optimization procedures were applied.

#### 3.1.2. Optimization

The first factor that should be considered during the GABA bioprocess is the concentration of MSG added in the fermentation culture. The researchers focusing on the production of GABA using *L. plantarum* FNCC 260 noted that increasing concentration of MSG in the culture broth switched to a significant increase in production [25]. MSG is a source of glutamate that was required as a precursor by the microorganism for the efficient production of GABA. The basic mechanism behind the increased production with increased MSG concentration was the induction of GAD enzyme production. Therefore, increasing the concentration of MSG increased the production of GAD, which was required for converting glutamate to GABA [28]. However, the concentration of the added MSG needed to be closely monitored. The concentration of MSG at 270 mM was found suitable to induce maximum biosynthesis. Increasing the concentration beyond this level increased the osmotic pressure and thus reduced the production of GABA.

Temperature was also an important physiological factor that needed monitoring to optimize the production. The study, using *L. brevis* CRL 1942, demonstrated high production of GABA at 30 °C. The report concluded that 22, 25, and 37 °C were unsuitable of for the production process. The conclusion on the right temperature to optimize production was credited to its effect on activity of GAD enzyme. The time factor also needs to be considered for optimal consumption of MSG—the study on *L. brevis* CRL 1942 demonstrated that its conversion rate was highest (90%) at 48 h of culture. The change in production rate was linked to changes in pH, caused by the release of hydrogen ions after decarboxylation of MSG to GABA. The higher concentration of hydrogen ions in medium caused a drop in pH, which could have affected the activity of GAD enzyme [18].

### 3.2. Lactobacillus Plantarum

#### 3.2.1. Production and Factors

Studies on Indonesian foods—tape ubi, gatot, growol, and bekasam—confirmed them to contain significant numbers of *Lactobacillus* species, which could be used to produce considerable amounts of GABA. With such a possibility, researchers isolated active strains from fermented food to study their ability to produce this compound. In the screening process, 30 *Lactobacillus* strains were isolated from above mentioned fermented foods [25]. These isolates were cultured in MRS broth at 37 °C for 24–48 h. MSG was added at a concentration of 118 mM in the growth medium, and a micro-aerophilic condition was maintained during the incubation of the cultures. The synthesis of GABA was detected for its presence in the culture filtrate, obtained from respective *Lactobacillus* species, and compared with pure form of a commercial GABA as a standard, using a method of thin layer chromatography. The best producer strains were identified by 16S rDNA sequencing and proteomic identification, using MALDI-TOF MS techniques. [25].

The identified isolates were further studied and two strains producing a significant amount of GABA were finally selected for GABA synthesis. *Lactobacillus plantarum* FNCC 260 and *L. plantarum* FNCC 434, under optimized conditions, produced an average of 352 mg/L and 328 mg/L of GABA, respectively. The synthesis timeline for maximum production of GABA was studied, the results obtained stated that the production started after 12 h of culturing of *Lactobacillus* strains, which was related to the start of cultures’ stationary phase. The synthesis of GABA increased after 48 h, and yield reached maximum after 60 h (809.2 mg/L) [25]. After this time, the concentration of GABA in culture started weakening. This decline was associated with production of enzyme GABA–transaminase (GABA–T E.C.2.6.1.19.), which catalysed the degradation of GABA molecule. The study suggested that genetic modification of the strains could help to reduce GABA–T production and thus the production of GABA could be prolonged from *L. plantarum* FNCC 260 strain.

Similar to other studies conducted with *L. brevis* strain [18,20], researchers also noticed a significant drop in pH during GABA production in *L.plantarum*. Within first 12 h, the pH dropped from 6.5 to 4.1. Unlike in *L. brevis*, where the drop in pH was recognized due to increased production of hydrogen ions in the decarboxylation process, in this case, the formation of acetic and lactic acid caused the drop in pH. It was also noted that despite the expression of GABA–T after 60 h of fermentation, the pH of the medium continued to decrease until 84 h, suggesting GAD was also active and GABA production was continuing.

#### 3.2.2. Optimization

Cofactors are essential molecules that help in the catalytic process. Therefore, various enzymes require certain cofactors to improve their efficiency. Addition of PLP in the medium has been reported to increase GAD activity, and subsequently, the production of GABA. In this case, adding 0.2 mM of PLP increased production to 903.0 mg/L, while 0.6 mM PLP increased the yield to about 945.3 mg/L [25]. These values were obtained after the cultivation of strains for 108 h.

Another interesting characteristic was noted that the production time was prolonged after the addition of PLP. In medium without supplementation of PLP, GABA production declined after 72 h; while in the presence of cofactor, the production was extended to 108 h. This finding was also supported by other studies performed using different GABA-producing strains. The addition of 0.1 mM pyridoxine also led to a significant increase in GABA production; therefore, this cofactor can also be used as an alternative additive to the culture media. The advantage of these cofactors is that they do not affect biomass production. The results suggested that the addition of the right amount of PLP could boost the production of GABA on larger scale [23].

The presence of MSG in medium was also noticed to be essential for microbial production of GABA using *L. plantarum* FNCC 260. The addition of 10 mM of MSG to *L. plantarum* culture medium increased production to 1226 mg/L after 96 h of fermentation. Beyond this time, glutamate production declined due to the expression of GABA–T, which synthesized succinic semi-aldehyde (SSA) from GABA [26]. Excess addition of MSG caused a decline in the production of GABA. The main reason for the decline was the toxic effect of higher levels of MSG on LAB strains. A higher concentration of MSG induced its toxic effect by suppressing *gad B* genes, which code for GAD [25].

Recombinant techniques were also applied to maximize production. The recombination increased the effectiveness of a bacterial strain, by improving its tolerance to acidic pH, caused by production of acetic and lactic acid, and the release of hydrogen ions. The genetic modification of the strain also reduced fermentation time. In a study, the recombinant *L. plantarum* FNCC 260 strain produced about 6450 mg/L of GABA within first 6 h. Besides this, the GAD activity was also above 73% after 6 h, which suggested the possible cause of higher yield [25] by the recombinant strain. The genetically modified strains are reported to reduce the production of GABA–T and other GABA-degrading enzymes. Therefore, genetic engineering can be an effective tool for increasing the production of GABA by several folds, through reducing the degradation of product, increasing the resistance for acidic pH, and shortening the production time. In one of such efforts, reverse genetics mechanism was used to identify the role of GABA metabolism in *Stagonospora nodorum* [29]. Recombinant techniques were also used to improve the synthesis in a process employing *C. glutamicum* [30].

### 3.3. Streptococcus salivarius

#### 3.3.1. Production and Factors

The fermentation process was conducted, cultivating *Streptococcus salivarius* in a nutrient medium supplemented with peptone, beef extract, MSG, dibasic ammonium citrate, and other compounds. The production of GABA was improved by adjusting the pH after every 12 h using sodium hydroxide. The study showed that the intracellular concentration reached its maximum at 24 h, and the extracellular concentration was highest at 84 h. The production of GABA increased after the pH of the medium dropped to about 4.5–5.0, and a significant production of GABA was detected even after cell death [21].

#### 3.3.2. Optimization

A study assessed the favourable conditions for GABA production in *S. salivarius* subsp. *thermophilus* Y2, as the optimum temperature in range of 40–45 °C and pH 4.5; although, the optimum temperature for GAD was 34–37 °C, and its activity was significantly inhibited above 46 °C [21]. The researchers suggested a need to maintain the conditions within these ranges to improve production. This study also reported that the addition of MSG significantly increased GABA production by *S. salivarius*, though the excessive addition caused significant toxicity. In addition to this, PLP also stimulated the production of GABA. Although the yield from *S. salivarius* subsp. *thermophilus* Y2 was significantly high, it was lower compared to the amount produced by other lactic acid bacteria. A strain of *Streptococcus thermophilus* produced higher concentrations of GABA in fermented milk, highlighting a natural production method for functional food [22].

### 3.4. Bifidobacterium and Lactobacillus Strains

#### 3.4.1. Production and Conditions

Although GABA production has been assessed using bacteria, mostly isolated from fermented food materials, production by bacteria isolated from gastrointestinal tract had not been examined. In the study, the researchers obtained 135 bacterial strains of *Lactobacillus* and *Bifidobacterium*. These bacteria were isolated from samples of saliva and faeces of volunteers and cultured in MRS with 10% carbon dioxide, and the incubation temperature was maintained at 37 °C for 24–48 h. *Bifidobacterium* strains required MRS medium supplemented with 1% MSG, 0.05% cysteine, and a suitable carbon source. When provided with a sufficient level of MSG, 24 strains of the *Bifidobacterium* and 111 *Lactobacillus* produced significant amounts of GABA [23].

Lactobacillus species were identified as *plantarum* and *brevis*, confirming initial reports of other researchers. *Bifidobacterium* strains included *B. dentium*, *B. angulantum*, and *B. adololescentis*. Comparing efficiency, the average production in all strains was between 50–6000 mg/L. *L. brevis* produced higher yield (679 mg/L), compared with *L. plantarum* strains, with an average production of about 300 mg/L. *Bifidobacterium* strains were better producers (an average of 2500–6000 mg/L of GABA) than the *Lactobacillus* strains [23]. The research demonstrated that *Bifidobacterium* strains could be employed for the industrial production of GABA. Furthermore, studies on GAD genes and their role in altering the production and the role of MSG as a precursor will allow the establishment of the optimum culture conditions needed for an economical commercial yield [28].

#### 3.4.2. Optimization

The manipulation of growth conditions optimized the shortest production time; where *Lactobacillus* strains produced the maximum amount of GABA after 72 h, and *Bifidobacterium* strains produced the maximum amount of GABA after 48 h [23]. The differences were associated with the different onset of stationary phases in growth cycle of both strains. In addition, PLP also had varying effects on bacterial strains. It was initially reported that the addition of PLP at the initial stages of fermentation had no significant impact on GABA production. However, adding this cofactor at the late stages of fermentation (after 72 h) led to improved production. The reason behind this increase was the late expression of the *gadB* gene, and the high degree of degradation of PLP during the lag and log phases of bacterial growth. For maximized production, it is important to use a fermentation method that will allow the addition of cofactor at the later stages of bacterial growth.

The cloning of genes from different strains through co-expression of GAD, derived from *Lactobacillus brevis*, also enhanced the production [30]. This approach minimized the degradation of PLP during the growth cycle of cultures. Bifidobacterium strains showed stable production of GABA due to the capacity of these strains to synthesize vitamin B6. Bifidobacterium strain can be genetically manipulated to overexpress the *gadB* and *gadC* genes and increase the expression of vitamin B6 synthesizing genes.

### 3.5. Monascus spp. and Rhizopus spp.

#### 3.5.1. Production and Factors

A few non-bacterial strains have also shown capability to produce significant amounts of GABA, one such reported organism is *Monascus sanguineus* [24]. The factors required to synthesize GABA by this mould were potato dextrose agar, magnesium sulphate, peptone, and incubation at temperature of 30 °C. The process time required for its cultivation was a minimum of one week under solid-state fermentation condition. Another species of fungi, *Monascus purpureus* CCRC 31615, was also found to produce GABA. The production of GABA compound was confirmed using chromatographic techniques.

A number of *Rhizopus spp* have been studied for biosynthesis of GABA under suitable conditions provided for fungal cultivation. One study assessed the effectiveness of *R. oligosporus* when it was grown using quinoa as a substrate, results demonstrated that the fungus produced up to 540 mg per kg of substrate used after 5 days of growth [26]. In addition, the production of significant amounts of vanillin acid, Gallic acid, and L-carnitine were also noted. A study using *R. Microsporus* var. *oligosporus* IFO 8631 reported that this strain could produce maximum amounts of GABA when fermented soybeans were added to the production medium. The duration of anaerobic and aerobic conditions in the fermentation process could also significantly affect the synthesis of GABA [27].

#### 3.5.2. Optimization

Techniques applied to optimize production of GABA by *Monascus spp* included the modification of growth conditions, substrate addition, growth time, and genetic manipulation. However, the production was also found to be dependent on the duration of fermentation. Maximum production by fungal cultures with 0.5% MSG addition was reported after 20 days [24]. Maintaining the right level of pH was also essential for maximizing the production of GABA by *Monascus spp*. *Monascus sanguineus* performed better with maintaining the pH of cultivation medium at 5.5. Furthermore, the wheat powder was the preferred carbon source by fungal cultures than the other substrates used, such as rice and sweet potato starch. A study on *M. purpureus* CCRC 31615 showed that addition of sodium nitrate increased GABA production to 1267.6 mg per kg of substrate used. Furthermore, the yield increased to 1493.6 mg/kg after the addition of dipotassium hydro phosphate. Improved production by *Rhizopus spp*. was achieved with implementation of optimized conditions of cultivation time, a suitable carbon source, and growth conditions [29].

## 4. Main Outcomes of Studied Bioprocesses

### 4.1. Requirements for Biosynthesis

The comparative study on processes employing bacterial and fungal strains showed that each microbial culture required its own specific factors to produce GABA. However, there were certain factors that were common among all microbes tested. Among these, the common factors were the supplementation of PLP and MSG, which are the same factors that have been reported in the study of GABA production employing *L. plantarum* FNCC 260 [25]. MSG acted as a precursor substrate, while PLP is a cofactor, required by enzyme GAD. The fermentation time varied between different strains. On average, most bacterial strains produced satisfactory amounts of GABA after 72 h. Others were able to prolong production time to more than 108 h, when optimized conditions were introduced to extend the stationary phase of GABA synthesis. Fungal strains required suitable solid particulate substrates as carbon sources for their mycelial cultivation under a solid state, along with both supplements of MSG and PLP. A fungal strain *Rhizosporus microsporus* var. *oligosporus* IFO 8631 was more effective under anaerobic conditions for GABA synthesis [27]. Therefore, the production of GABA using any of the strains would require a detailed study on their physiology and the GABA synthesis phase in their growth cycle.

### 4.2. Manipulations of Microbes

The analysis of studies conducted using various GABA-producing microorganisms revealed that strains could be manipulated to obtain better yield of this molecule. Some of the microorganisms were also genetically modified for the purpose [19]. Genetic engineering helped in improving production, boosting tolerance, and reducing the virulence under toxic conditions. One of the methods used in altering the genetic sequence of strains was to either activate or shut down specific pathways. The process can be performed either by the deletion or the silencing of respective genes, using this strategy, the performance of *Stagonospora nodorum* could be improved through its genetic modification [29]. The main objective of this molecular technique was to enable the microorganism to gain specific characteristics that would help increase GABA production. The maintenance of an acidic pH in fermentation medium was identified as a method of optimizing production, in all GABA producers [25,26,27,28,29,30,31]. The addition of PLP and MSG at their specific concentrations was also essential in boosting GABA production. Besides some common factors, microbial strains, specifically selected for GABA synthesis, might require additional requirements and modifications, which would be unique to each strain of bacteria or fungi for achieving maximum yield of GABA [32,33,34,35,36,37].

Studies on GAD genes have also been performed to assess their role in altering the production of GABA in different strains. An interesting study reported that the usage of recombinant GAD modified from *L. plantarum* FNCC 260, with expression through *Escherichia coli*, triggered a GABA yield 5-fold higher than that which was obtained in the fermentation process with the usual LAB process [19].

On sequencing the genes of the GABA-producing strains, the researchers identified that most of the strains expressed the *gadB* gene after the provision of sufficient measures of MSG. However, the mutations on the *gadB* gene in nine strains of *L. fermentum* was also noted, which explained why these strains did not synthesize any GABA, despite having the *gadB* gene. Other studies have also reported that mutations might have affected the production of GABA in several bacterial strains. Besides this, decreased compound production has also been linked with deletion or mutation of the *gadC* gene. The gene encodes for an antiporter that exports GABA out of the cells. Excessive accumulation of the compound inside the microbial cell could have a negative impact on the biosynthetic pathway [28]. Reverse genetics is also applied to manipulate microorganisms for maximum production. An example of reverse genetics is applied in creating genetic mutants lacking the Sdh1 gene that is required for succinic semialdehyde dehydrogenase production. That was confirmed by inserting this gene, which led to increased production of GABA by *Stagonospora nodorum* [29]. Such possibilities have further enhanced its significance as a commercial compound, which could be produced by microbes as a secondary metabolite. With the extensive information available as discussed above, GABA production could be developed for use in several industries, including nutraceuticals, food, and pharmaceutical [38,39,40,41].

### 4.3. Effective Strategies

The immobilization and multilayer co-encapsulation techniques for effective employment of selected microbes have been established for enhanced biosynthesis of GABA, through their use in multi-cycle processes [40,41,42]. In a study, an immobilized form of enzyme GAD was used for the biosynthesis of GABA, for immobilization first this enzyme was synthesized in fermentation process growing a LAB (*L. plantarum*) in the rice vinegar and MSG medium [43]. The *GadB* gene encoding GAD from *L. plantarum* was expressed in *Lactococcus lactis*. The characterization studies confirmed that the recombinant *GadB* was a homodimer, and its maximal performance was achieved at pH 5.0 and at its optimum temperature of 40 °C. The elevated production of GABA has been reported using an immobilized form of GAD. The support material for immobilization used for the immobilization of enzyme was a porous material—a hybrid of organic–inorganic amphiphilic [44].

The effective strategies enhancing GABA biosynthesis by specifically selected microbial strains should be tried for obtaining better yields, such as those in molecular cloning, expression and immobilization of GAD [45], and physiology-oriented engineering [46]. GAD system is highly unpredictable in different strains, as some species have one, two, or three GADs followed by antiporters either none or one, sometimes even two. For example, *Mycobacterium tuberculosis* has been reported to have a GAD gene without an antiporter [47]; however, *Listeria monocytogenes* has been reported to have three GADs and two antiporters [48]. Therefore, the properties of GAD at chemical and physical levels differentiate considerably in quite a few strains, hence, new GAD enzymes are continuously searched for, with a possibility of finding one with a higher biotechnological significance. GAD gene sequence detected in different microorganisms also can suggest its capability to produce GABA at genetic level [49,50]. In such approaches, *Lactococcus lactis* has been studied for improved output of a biosynthetic process [51].

Two isozymes of GAD have been studied in *Lactobacillus brevis* for their contribution in GABA synthesis, by providing resistance to acid formed in fermentation medium [52]. Some researchers have preferred to use those microbial strains for GABA biosynthesis, which were isolated from food sources such as cheese, sourdough, and fruits [53]. In the fermentation process, MSG, a GABA-precursor, was replaced by plant sources, such as fenugreek seeds and soybean, or an animal source such as gelatine, which contained glutamic acid or glutamine. In order to control similar amounts of either precursor in all fermentation batches, the amount of plant or animal sources containing the same concentrations of glutamine and glutamic acid as those in control MSG-supplemented experiments, were used [53].

The demand in several countries for foods enriched with GABA has increased to obtain the benefit of the physiological functions of this compound. However, some researchers have contradicted the direct addition of this compound to food items, stating their concern on this practice being unnatural and unsafe [54]. Therefore, alternative applications of GABA, other than as a food supplement, should be explored for medicinal purposes, such as in wound healing [55].

In this overview article, it is not possible to discuss all bioprocesses, therefore Table 2 has summarized some of the microorganisms selected to perform as biocatalysts in GABA production and their essential factors applied in the biosynthesis process. The references included in Table 2 have emphasis on certain requirements, which could be used and maintained during fermentation cycle to enhance the yield of GABA in microbial synthesis processes. The studies have also used supplementations, including inorganic nitrogen sources such as ammonium salts, organic sources such as peptone, casein, and beef extract, NaCl, initial MSG concentration, required amount of GAD-cofactor PLP, etc. The results concluded that the selection of ingredients, constituting a fermentation medium, with specifically selected sources of carbon, nitrogen, and other components should be optimised for improved yield of GABA. Using such an approach, *L. lactis* was cultivated in a nutritional medium using a mixture of three carbon–nitrogen sources at a ratio of 33:58:9, including brown rice juice, germinated soybean juice, and enzymolyzed skim milk, which produced a good yield of up to 6.4 g/L [56].

Studies have also suggested the use of selective culture cultivation systems for operating the process of biosynthesis, depending on type of microorganism/s being employed, such as a batch, fed-batch, single-culture, or co-culture in liquid-submerged fermentation. The effectiveness of solid-state fermentation system should be exploited, when employing mycelial–fungal strains on solid substrates. Columns 2 and 3 in Table 2 include the details of such factors and process requirements.

## 5. Conclusions

The information presented in the above sections has clearly identified the fast-growing potential of GABA in health sector, due to its several pharmacological properties. Therefore, with an increasing demand in the market, there is a need for establishing an effective biosynthetic process for its availability at a lower cost. This review has summarized information on specific microorganisms involved in GABA production, aiding understanding of their requirements for essential factors needed in their growth cycle to achieve the optimum synthesis GABA. Suitable methods could be used for optimizing the yield in a cost-effective process. This is a concise overview only providing selected information on the biosynthesis of this compound. Future studies can focus on improving production by genetic manipulation to enable some of the strains to reduce the need to add the PLP compound in the production medium. The target would be aimed at reducing the cost of production of GABA on a large scale, through a microbiological route. The study of efficient biocatalysts and their factors required in microbial synthesis of GABA will certainly help to establish a yield-efficient, low-cost production process. This approach will ensure its availability as a low-cost ingredient for application in food and pharmaceutical industries.

## Figures and Tables

**Table 1:** Commercial GABA Products Available in Market **.

Brand Names	Form of Supplements	Route of Synthesis (Microbial/Chemical)	Commercial Producers
Now supplements	500 mg capsules	not known	Vitamin Angels
Double Wood Supplements	1000 mg capsules	not known	Double Wood Supplements
Natural source GABA	Gelatin capsules	not known	Pure Organic Ingredients
Horbäach GABA	500–1000 mg capsules	not known	Horbäach
GABA calm	Tablet	not known	Source Naturals
Liposomal GABA	Liquids	not known	Quicksilver Scientifics
Amazing Formulas GABA	500–1000 mg GABA	not known	Amazing and Nutrition

** Information sourced from: https://www.amazon.com/gaba/s?k=gaba (accessed on 26 November 2021).

**Table 2:** Selected Microbial Systems and Their Requirements for the Synthesis of GABA.

Microorganisms Employed in Bioprocess	Yield of GABA, or Product	Requirements	Reference
*Lactobacillus brevis* CRL 1942, an isolate from quinoa sourdough	50 mM after 96 h	MSG 53 mM PLP, pH-4.5, 30 °C	[18]
*Escherichia coli* *BL21(DE3)/pET32a-gadA*, BL21(DE3)/pET32a-*gadAB*, BL21(DE3)/pET32a-*gadABC*	23.6 g/L at 36 h in batch fermentation 31.3 g/L at 57 h in fed batch fermentation	Engineered MSG pathway 3 genes *gadA*, *gadB*, *gadC* cloned	[19]
*L. brevis* GABA 057 by gel-entrapment of bacterial cells	223 mM after 48 h of fermentation	534 mM MSG; addition of iso-malto oligosaccharide to alginate beads, 34 °C, pH 4.2	[20]
*S. salivarius* subsp. *thermophilus* Y2	7984.75 ± 293.33 mg/L at 48 h	PLP 0.02 mmol/L Peptone, beef extract, MSG, ammonium citrate, 40–45 °C, pH 4.5–5.0	[21]
*Streptococcus thermophilus*, co-culture with *L. rhamnosus* production of fermented milk enriched with GABA	5.4 g/L; 8.3 g/L when co-cultured with *L. rhamnosus;*	1 g/L skim milk powder, or yeast extract or soy protein hydrolysate 10 g/L MSG at 37 and 42 °C	[22]
*Lactobacillus* and *Bifidobacterium* sp isolated from human microbiota	*L. brevis* 675 mg/L, *L. plantarum* 300 mg/L Bifidobacteria 2500–6000 mg/L	MRS, 1% MSG, 0.05% cysteine. 37 °C PLP (some synthesised Vit-B6, needed no PLP supplement)	[23]
*Monascus sanguineus*, a fungal species belonging to family Elaphomycetaceae	15.53 mg/gm dry substrate (20 days solid-state fermentation).	Wheat powder, potato dextrose, magnesium sulfate, peptone, 30 °C. MSG 0.5%, pH-5.5	[24]
*L. plantarum* FNCC 260, isolates of Indonesian fermented foods	945.3 mg/L at 108 h	MSG, MRS medium 0.1 mM PLP, 37 °C, pH 4.1	[25]
*Rhizopus oligosporus*	540 mg/kg (50 h) in fermented foods by solid-state fermentation	Quinoa, soybeans pH-5.5, PLP, MSG-0.5%	[26,27]
*Escherichia coli* genetically engineered with *StGAD*, *SsGAD*, and *ScGAD* used as whole-cell biocatalysts	2.771 kg/L with average molar conversion rate of 67% in 20 h by glutamate decarboxylation of MSG/L-glutamic acid	GAD from Streptomyces; engineered *E.coli* cells were repeatedly used for 10 times, 37 °C	[28]
*Corynebacterium glutamicum* recombinant strain with co-expression of 2 LAB-genes	22.57 ± 1.24–30.18 ± 1.33 g L⁻¹ 36 h	*gadB*1 and *gadB*2 from *L. brevis;* urea supplementation	[30]
*Streptococcus thermophilus* APC151, isolate of fish digestive tract	Bioactive yogurt enriched with 2 mg/mL GABA produced in fermentation	14% (*w*/*v*) skim milk; 2.25 mg/mL MSG; 42 °C for 48 h	[31]
*L. Plantarum* Taj-Apis 362 overexpression	11.09 mM in 60 h; predicted value 11.23 mM by response surface methodology	GAD enzyme; 497.973 mM glutamic acid, 36 °C, pH 5.31	[37]
*L. plantarum* EJ2014	19.8 g/L 159.7% of theoretical maximum yield	100 g/L yeast extract, 10 g/L glucose, 2.25% MSG	[39]
Probiotic bacterial strains	Development of functional food products	Co-encapsulation of bacterial cells with bioactive compound in a single matrix	[40]
Lactic acid bacteria	Microcapsules of LAB and GABA for targeted intestinal delivery in functional food formulations	Dextran, whey protein, multilayer co-encapsulation, two-stage ultrasonication	[41]
*L. plantarum* co-microencapsulation with thermostability and biocompatibility	Multifunctional food microcapsules containing GABA, probiotics, and prebiotics	Matrix of exo-polysaccharides dextran, inulin, maltodextrin	[42]
*L.s plantarum*	Rice vinegar MSG with food-grade γ-aminobutyric acid	Immobilized enzyme GAD from *L*. *plantarum*	[43]
*L. fermentum* YS2, expressed in *E. coli* immobilized CBM-GAD catalyzed GABA synthesis	Yield 5.15 g/L; productivity 3.09 g/L per hour by immobilized enzyme for 10 repeated uses	GAD molecular cloning, expression, immobilization	[45]
*gadA*, *gadB*, *gadC*, *gadCB*, *gadCA* from *L. brevis cloned into pMG36e*	43.65 g/L at 98.42% GABA conversion rate	Glucose yeast extract peptone medium, engineering of bacterial cells for physiology orientation	[46]
*Lactococcus lactis* subsp. lactis CV56 co-expression of *gadC* and *gadB*	25.61 g/L in a two-stage pH control batch fermentation strategy	50 °C, pH 4.7 genetic engineering of *Escherichia coli* cells	[52]
*L. brevis* 9530: pNZ8148-*gadBC* isozymes of glutamate decarboxylase	104.38 ± 3.47 g/L at 72 h fed-batch fermentation with two-stage pH and temperature control	Overexpression of GAD isoforms of GAD, *gadA*, and g*adB*	[53]
*L. delbrueckii* subsp*. Bulgaricus;* bacterial isolate of Encián cheese sourdough; Pseudomonad and Enterobacteria isolates of banana	90% activity; 87.8%; 88.2%	MSG 1.3 mg/mL, NaCl 2%, amount of PLP:MSG 0.72:1.3, pH 6.5, 33 °C	[54]
*L. plantarum* DSM19463	4.83 mM of GABA; grape beverage as a functional drink; dermatological application	grape must, addition of 18.4 mM L-glutamate, pH 6.0, 30 °C, fermentation 72 h.	[57]
*L. brevis* NCL912 response surface methodology	1005.81 ± 47.88 mM, in 48 h fed-batch fermentation	30–35 °C, 5.0 and 250–500 mM glutamate	[58,59]
*L. brevis* RK03	62,523 mg/L, after 88 h	1% glucose; 2.5% yeast extract; 2 ppm CaCO₃, MnSO₄, Tween 80; 10 μM PLP, 650 mM MSG	[60]
*L. brevis* NCL912 10 litre fermenter at 100 rpm mixing	205.8 ± 8.0 g/L after 48 h of fermentation MSG replaced by glutamic acid	295 g/L glutamic acid, 25 g/L each glucose and yeast extract; 25 mg/L MnSO_4_·H_2_O; 2 g/L Tween-80; 32 °C	[61]
